# Hybrid Harmony: A Multi-Person Neurofeedback Application for Interpersonal Synchrony

**DOI:** 10.3389/fnrgo.2021.687108

**Published:** 2021-08-12

**Authors:** Phoebe Chen, Sophie Hendrikse, Kaia Sargent, Michele Romani, Matthias Oostrik, Tom F. Wilderjans, Sander Koole, Guillaume Dumas, David Medine, Suzanne Dikker

**Affiliations:** ^1^Psychology Department, New York University, New York, NY, United States; ^2^Department of Clinical Psychology, Vrije Universiteit Amsterdam, Amsterdam, Netherlands; ^3^Methodology and Statistics Research Unit, Institute of Psychology, Leiden University, Leiden, Netherlands; ^4^Electrical Engineering, Mathematics and Computer Science Department, University of Twente, Enschede, Netherlands; ^5^Independent Researcher, Amsterdam, Netherlands; ^6^Department of Psychiatry, Centre Hospitalier Universitaire Sainte-Justine Research Center, University of Montreal, Montreal, QC, Canada; ^7^Mila – Quebec Artificial Intelligence Institute, University of Montreal, Montreal, QC, Canada; ^8^Diademics Pty Ltd., Mount Waverley, VIC, Australia; ^9^New York University-Max Planck Center for Language, Music, and Emotion, New York University, New York, NY, United States

**Keywords:** hyperscanning, neurofeedback, brain-computer-interface, EEG, inter-brain coupling, real-world neuroscience

## Abstract

Recent years have seen a dramatic increase in studies measuring brain activity, physiological responses, and/or movement data from multiple individuals during social interaction. For example, so-called “hyperscanning” research has demonstrated that brain activity may become synchronized across people as a function of a range of factors. Such findings not only underscore the potential of hyperscanning techniques to capture meaningful aspects of naturalistic interactions, but also raise the possibility that hyperscanning can be leveraged as a tool to help improve such naturalistic interactions. Building on our previous work showing that exposing dyads to real-time inter-brain synchrony neurofeedback may help boost their interpersonal connectedness, we describe the biofeedback application Hybrid Harmony, a Brain-Computer Interface (BCI) that supports the simultaneous recording of multiple neurophysiological datastreams and the real-time visualization and sonification of inter-subject synchrony. We report results from 236 dyads experiencing synchrony neurofeedback during naturalistic face-to-face interactions, and show that pairs' social closeness and affective personality traits can be reliably captured with the inter-brain synchrony neurofeedback protocol, which incorporates several different online inter-subject connectivity analyses that can be applied interchangeably. Hybrid Harmony can be used by researchers who wish to study the effects of synchrony biofeedback, and by biofeedback artists and serious game developers who wish to incorporate multiplayer situations into their practice.

## Introduction

What does it mean to lose yourself in someone else? How is it possible that the mere physical presence of another human can make us believe we can conquer the world, or conversely, make us feel lonely and incapable? We know, both scientifically and intuitively, that relationships are crucial for our physical and mental well-being (Pietromonaco and Collins, 2017). But they are also sources of frustration in their fluid, messy mix of internal inconsistencies: love and hate, inclusion and exclusion, fascination and comfort, challenge and familiarity. Can we capture this seemingly subjective, fleeting, and elusive notion of “being on the same wavelength” with another person, with objective measurement tools? And if so, can we leverage this information to guide people in their interaction with others?

Successful social interactions require tight spatiotemporal coordination between participants at motor, perceptual, and cognitive levels. Around a decade ago, several labs began to use a variety of methods to record (neuro)physiological data from multiple people simultaneously, a technique known as “hyperscanning.” This has allowed researchers to study dynamic coordination in a range of social situations such as ensembles performing music, multiple people performing actions together, or carrying on a conversation (Babiloni et al., 2006; Tognoli et al., 2007; Dumas et al., 2010; Yun, 2013; Zamm et al., 2018b; see e.g., Hari et al., 2013; Babiloni and Astolfi, 2014; Czeszumski et al., 2020 for reviews). There now exists a growing body of work where pairs or groups of participants engage in social interactions while their brain activity, physiological responses, and (eye) movements are monitored.

**Figure 1:**
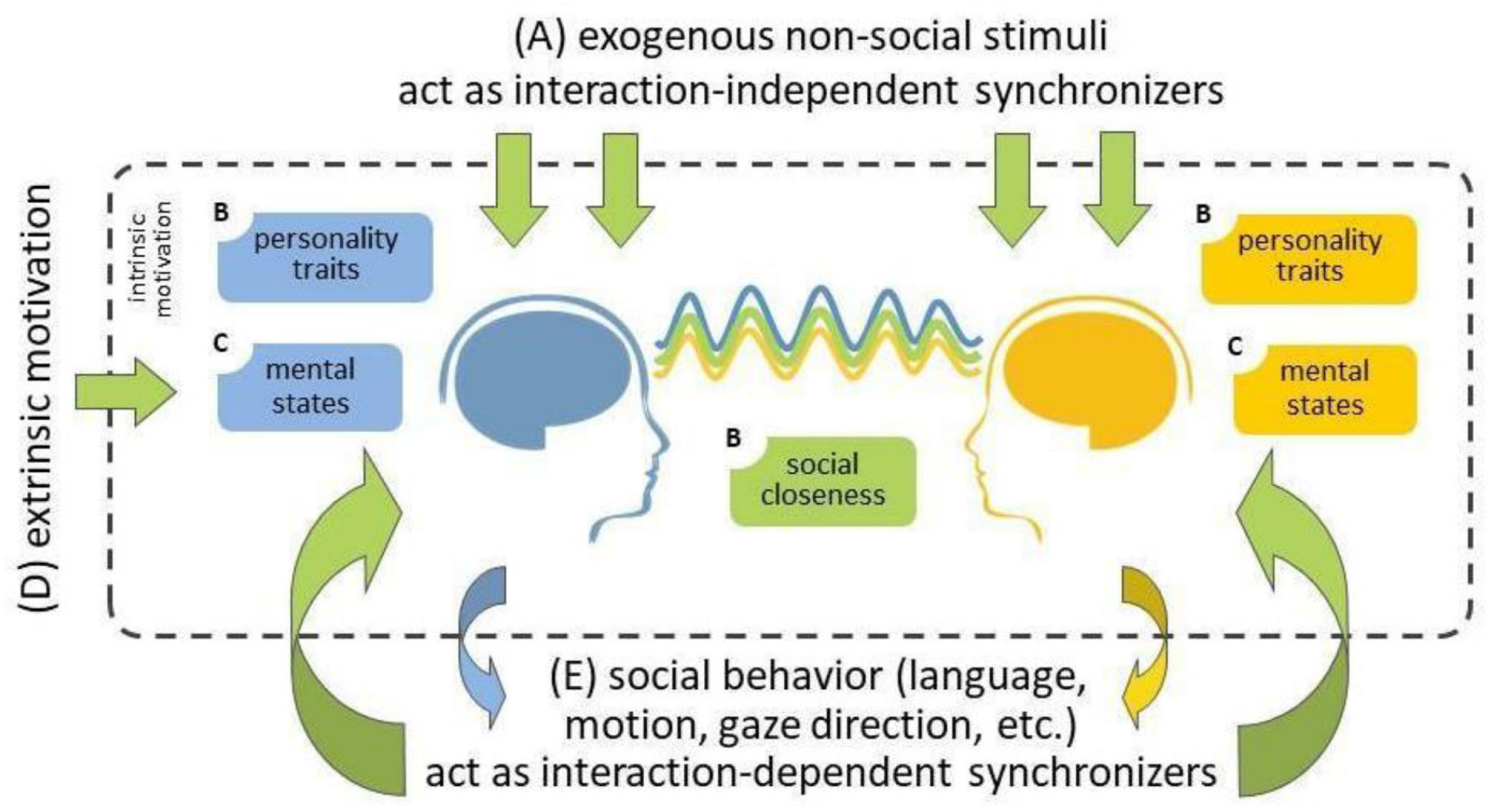
A summary of possible sources of inter-brain synchrony during social interaction (adapted from Dikker et al., 2021). **(A)** External non-social stimuli (top) and **(E)** social behavior (bottom) provide exogenous sources of shared stimulus entrainment and interpersonal social coordination, respectively, leading to similar brain responses, i.e., inter-brain synchrony. **(B)** Both individuals' social closeness and personality traits (e.g., affective empathy) affect their social engagement during the interaction, and thus the extent to which their brain responses become synchronized. **(C)** participants' mental states (e.g., focus) similarly affect participants' engagement with each other, intrinsically (endogenously) motivate participants to make an effort to connect to each other. **(D)** Such engagement can be “boosted” via extrinsic motivation, which could subsequently lead to increased inter-brain synchrony.

While hyperscanning research is also conducted using hemodynamic neuroimaging tools, including functional Magnetic Resonance Imagining (fMRI; Koike et al., 2016, 2019; Abe et al., 2019) but especially functional near-infrared spectroscopy (fNIRS; Scholkmann et al., 2013; Nozawa et al., 2016; Reindl et al., 2018), we here focus on electroencephalography (EEG) hyperscanning. The extent to which EEG activity becomes synchronized between people is correlated with a range of factors. For example, it has been widely demonstrated, in both single-brain laboratory research and hyperscanning studies, that shared attention to the same stimuli leads to similar brain responses across individuals, and consequently, higher inter-brain synchrony (Figure 1A; Hasson, 2004; Dikker et al., 2017; Czeszumski et al., 2020). Importantly, social behavior has also been shown to serve as an (exogenous) source of interpersonal synchrony (Figures 1B,E): Behaviors such as joint action, language, eye contact, touch, and cooperation drive synchrony in various social contexts (Dumas et al., 2010; Dikker et al., 2014, 2021; Kinreich et al., 2017; Goldstein et al., 2018; Pérez et al., 2019; Reinero et al., 2021). Furthermore, both individuals' social closeness and personality traits (e.g., empathy) affect people's social engagement during an interaction, and thus the extent to which their brain responses become synchronized (Dikker et al., 2017, 2021; Kinreich et al., 2017; Goldstein et al., 2018; Bevilacqua et al., 2019). Participants' mental states (e.g., focus) similarly influence participants' engagement with each other, endogenously motivating them to make an effort to connect to each other (Figure 1C; Dikker et al., 2017, 2021).

Importantly, people can also be extrinsically motivated to socially engage with each other (Figure 1D). Specifically, our group recently reported that exposing people to a hyperscanning neurofeedback environment can motivate social engagement (Dikker et al., 2021). Using data from the interactive social neurofeedback installation The Mutual Wave Machine (wp.nyu.edu/mutualwavemachine), we show that dyads who were explicitly made aware of the social relevance of the neurofeedback environment, exhibited an increase in inter-brain coupling over time. This suggests that external factors may help boost interpersonal engagement, which raises the possibility that interpersonal synchrony biofeedback may be one fruitful avenue to pursue in such efforts.

However, while neurofeedback applications using data from individual brains are fairly widely used across scientific, clinical, educational, and artistic contexts (see e.g., van Hoogdalem et al., 2020 for a review), to our knowledge multi-person neurofeedback using hyperscanning EEG has been implemented primarily in game and art environments (see contributions in Kovacevic et al., 2015; Dikker et al., 2019, 2021; Nijholt, 2019; see Duan et al., 2013; Salminen et al., 2019 for examples of scientifically oriented dual-brain neurofeedback experiments). As a result, little is known about the possible effectiveness of hyperscanning neurofeedback in improving social communication.

This is further complicated by the fact that consensus is lacking with regard to how synchrony should be computed (Ayrolles et al., 2021). While some metrics have been shown to be “better” than others from a purely statistical perspective (Burgess, 2013), only very few scholars have attempted to map computational choices with regard to interpersonal neural connectivity to psychological processes or constructs (Dumas and Fairhurst, 2021; Hoehl et al., 2021). This distinguishes synchrony neurofeedback from other BCI applications, such as so-called “P3 spellers” (Fazel-Rezai et al., 2012), which are based on well-established neural signatures.

Because of the lack of consensus with regard to optimal synchrony metrics, we argue that it is desirable that multi-brain neurofeedback applications allow users to select from various synchrony metrics that can be used independently, and thus explore the utility of different metrics in different contexts.

To this end, we have developed Hybrid Harmony, a Brain-Computer Interface (BCI) that uses a hyperscanning approach to allow the collection of EEG data from two or more people simultaneously and enables users to visualize/sonify the extent to which participants' biometrics are coupled, choosing between different synchrony metrics. These metrics, described in section Connectivity Analysis, are developed in parallel with HyPyP, an open-source Python-based pipeline that allows researchers to compute and compare different inter-brain connectivity metrics on the same dataset (Ayrolles et al., 2021).

## Software Description

### Overview

**Figure 2:**
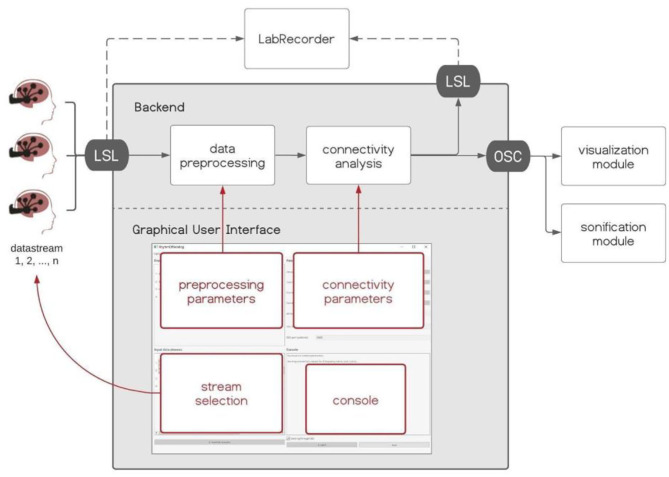
Hybrid Harmony software. The backend and Graphical User Interface (GUI) of Hybrid Harmony are shown in the gray box. Hybrid Harmony performs data preprocessing and connectivity analysis on the incoming EEG data from LabStreamingLayer (LSL), and outputs synchrony values to LSL and Open Sound Control (OSC; detailed in Supplementary Material, The Data Transfer Protocol: Open Sound Control). The output can then be recorded by LabRecorder (Supplementary Material, Saving Data Through LSL) and be transformed into sensory experiences through the visualization and sonification modules (section Visualization and Sonification). The GUI enables the user to control parameters for preprocessing and connectivity analysis, as well as monitor the program status on the console.

Hybrid Harmony is an open-source software package written in Python (https://github.com/RhythmsOfRelating/HybridHarmony), accompanied by a visualization module and a sonification module (Figure 2). The software consists of a backend that handles data acquisition and performs analyses, and a Graphical User Interface (GUI) made with PyQt5 (https://www.riverbankcomputing.com/software/pyqt/), where users can control parameters for the analyses (Figure 2). We introduce the software and discuss compatible hardware (EEG systems) in section Hardware, the processing pipeline including preprocessing and connectivity analysis in section Data Preprocessing, Connectivity Analysis, Normalization, and the visualization and sonification modules in section Visualization and Sonification. Data transfer protocols, i.e., LabStreamingLayer (LSL, https://github.com/sccn/labstreaminglayer) and Open Sound Control (OSC, Wright and Momeni, 2011), are described in Supplementary Material, Data Transfer Protocol. Detailed instructions can be found on the GitHub page.

### Hardware

The tool is compatible with any EEG device that interfaces with LabStreamingLayer, and has been tested with MUSE (https://choosemuse.com/; Bhayee et al., 2016), emotiv (EPOC and EPOC+; https://www.emotiv.com/; Williams et al., 2020), the SMARTING system from mBrainTrain (https://mbraintrain.com/; Grennan et al., 2021), and Brain Vision LiveAmp systems (https://www.brainproducts.com/; Fang et al., 2019), and can be expanded to accommodate other systems that export data to LabStreamingLayer.

### Data Preprocessing

The first stage of data preprocessing is a buffering procedure that holds and segments incoming data time-series. Incoming streams from LSL are stored in a 30 s buffer updating at the EEG data's sampling rate (e.g., 250 Hz for Brain Vision LiveAmp system). Hybrid Harmony then selects the most recent time window to perform the signal processing procedure. The time window is determined by “window size” and is 3 s by default. The rate of the analysis depends on the computation bandwidth of the system Hybrid Harmony is running on. For example, on many systems we tested data are analyzed roughly 3.5 times per second.

The time window is filtered with the infinite impulse response (IIR) filter (Oppenheim, 1999) into frequency bands of interest (e.g., 8–12 Hz for the alpha frequency band), and then Hilbert transformed to generate the instantaneous analytic signal (Oppenheim, 1999). Users can choose to output spectral power concurrently by selecting the “sending power values” checkbox: power spectral density will be computed and sent to LSL along with connectivity values.

**Figure 3:**
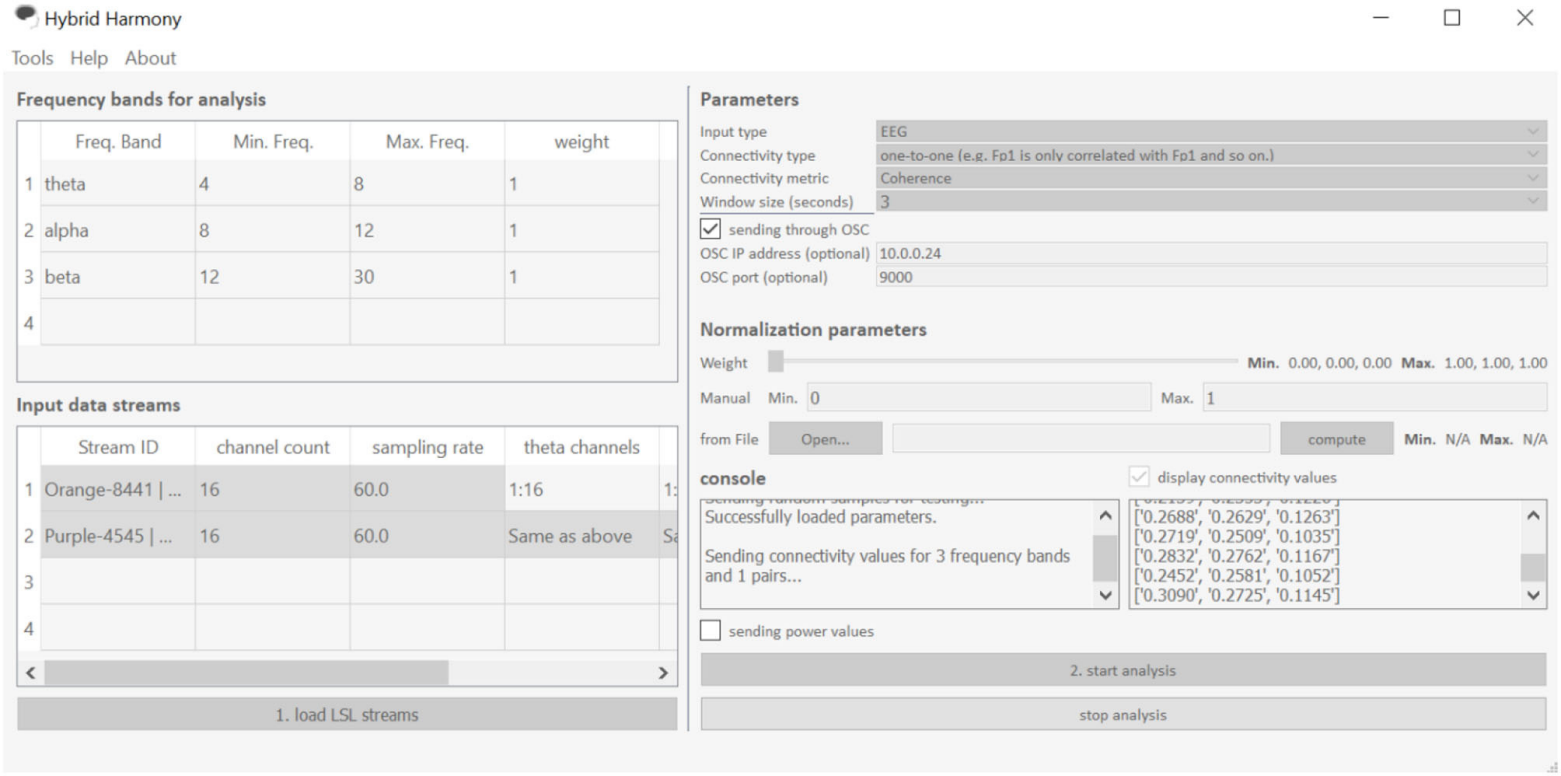
The Hybrid Harmony GUI. The interface is divided into five sections: “Frequency bands for analysis,” “Input data streams,” “Parameters,” “Normalization parameters” and “Console,” the first four of which allow users to specify the parameters detailed in section Data Preprocessing, Connectivity Analysis, Normalization. The GUI facilitates three main actions shown as buttons “load LSL streams,” “start analysis,” and “stop analysis.” “Load LSL streams” will start the stream discovery (Supplementary Material, Stream Discovery Through LSL) in the backend; “start analysis” button initiates the analyses (section Connectivity Analysis and Normalization) and data transferring (Supplementary Material, Using LSL for Output). “Stop analysis” will pause the analyses and allow the parameters to be edited.

**Figure 4:**
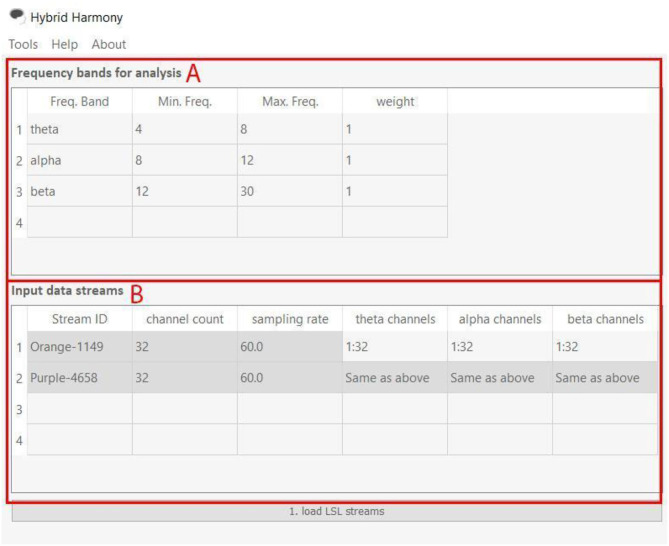
Frequency bands for analysis and Input data streams of the Hybrid Harmony GUI. The left half of the GUI, i.e., the “Frequency bands for analysis” and “Input data streams” tables control parameters for data preprocessing (section Data Preprocessing). **(A)** “Frequency bands for analysis” has four editable columns: Freq. Band: Name of the frequency band; Min. Freq.: The lower bound frequency for the band; Max. Freq.: The upper bound frequency for the band; weight: Weighting factor of the current band (connectivity values will be multiplied by this factor). **(B)** “input data streams” has three non-editable columns: Stream ID: Name of the EEG stream; channel count: number of EEG channels; sampling rate: sampling rate of the EEG streams. It also has editable columns, corresponding to frequency band names that users typed in **(A)**. The columns theta channels, alpha channels, and beta channels thus determine channel indices to use for computing connectivity values.

The GUI allows users to change processing parameters via “Frequency bands for analysis,” “Input data streams,” and “window size” (Figure 3). “Frequency bands for analysis” is an editable table specifying the frequency bands of interest, where “Freq. Band” denotes the frequency band name, “Min. Freq” and “Max. Freq” denote the lower and upper bounds of the frequency band, and “weight” determines how the different bands are weighted relative to one another. Figures 3, 4 show the default setup for this table. “Input data streams” displays the name, channel count, and sampling rate of the incoming streams, and its editable cells (e.g., “theta channels,” “alpha channels,” etc.) determine the specific channels to use for each frequency band. Lastly, the “window size” text field determines the length of the data segment for the analysis.

### Connectivity Analysis

**Figure 5:**
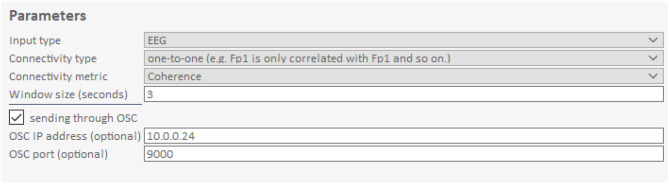
Connectivity parameters of the Hybrid Harmony GUI. The upper right part of the GUI, i.e., the “Parameters” control parameters for connectivity analysis (section Connectivity Analysis). Input type specifies the type of data (here: EEG). Connectivity Type determines how the connectivity values are averaged across electrode pairs. Connectivity metric determines the calculation of synchrony between two signals. Window size is the length of the data segment to compute synchrony over online. The checkbox sending through OSC determines whether connectivity values are sent through OSC (Supplementary Material, The Data Transfer Protocol: Open Sound Control) in addition to LSL. OSC IP address and OSC port are used to transport data.

Connectivity Analysis takes the analytic signal from Data Preprocessing as input, and computes one connectivity value for each electrode pair per frequency band in every participant pair. Then, connectivity values are averaged across electrode pairs, so the output is one connectivity value per frequency band for every participant pair. The exact computations are adapted from the python-based inter-brain analysis pipeline HyPyP (Ayrolles et al., 2021), and listed in Table 1. The user may choose from a list of connectivity measures by changing the “Connectivity metric” parameter (Figure 5). Currently implemented metrics include coherence, imaginary coherence, envelope correlation, power correlation, phase-locking value (PLV), and Circular Correlation Coefficient (CCorr). The mathematical equations and references of these metrics are provided in Table 1. In the equations, *X*(*i, t*) denotes the analytic signal for subject *x* at channel *i* for time point *t*, and *Y*(*j, t*) is that for subject *y* at channel *j* for time point *t*, and the star sign denotes the complex conjugate. The result of each equation is one synchrony value per electrode pair, written as *r*_*i, j*_, and the computation is carried out for all electrode pairs (*i, j*) where *i* belongs to subject *x* 's channels and *j* belongs to subject *y* 's channels. Note that we are computing all metrics using the analytic signal from the previous step to streamline the computation. We compute synchrony values from the analytic signals using Hilbert Transform (HT), an alternative analysis to the windowed Fast Fourier Transform (FFT). The analytic signal from HT and the spectra from the windowed FFT both represent the amplitude and phase of the signal in their real and the imaginary parts, respectively, except that the analytic signal is “instantaneous,” while the windowed FFT is an average value over a period (Kovach, 2017). Therefore, while the cross-spectra in coherence is usually the expected value of the product between the two signals' spectra, we used a computation (Table 1) adapted from Equation (1) (Kovach, 2017), i.e., the cross-spectra are expressed as the expectation of the dot product between *X*(*i, t*) and the complex conjugate of *Y*(*i, t*). Using this formulation is appropriate when investigating the synchronization of signals as it allows us to measure their similarity on a sample-by-sample basis, not just as an average over a relatively long time-window.


(1) $$\begin{array}{l} \hat{S_{xy}}=\frac{1}{T}\underset{T}{\int }X\left(t\right){Y\left(t\right)}^{*}dt \end{array}$$

Currently, only one metric at a time can be employed, but a user can run multiple instances of the software and thus output multiple metrics simultaneously. The user can then record these streams using LabRecorder (Supplementary Material, Saving Data Through LSL). For visualization, it is possible to differentiate the streams based on the unique source_id in the metadata of the LSL stream, and choose only one to display. However, this feature is not yet developed in our visualization module.

**Table 1:** Connectivity metrics adapted from Ayrolles et al. (2021).

**Connectivity metrics**	**Equation**	**References**
Coherence	$r_{i,j}=\frac{\left|{X\left(i,t\right)\cdot Y\left(j,t\right)}^{*}\right|}{\;\sqrt{\overset{T}{\underset{1}{\sum }}|X\left(i,t\right){|}^{2}\cdot \overset{T}{\underset{1}{\sum }}|Y\left(j,t\right){|}^{2}}}$	Guevara and Corsi-Cabrera, 1996; **Dikker et al.**, **2017**
Imaginary coherence	$\;r_{i,j}=\frac{\left|\text{imag}(X(i,t)\cdot Y{\left(j,t\right)}^{*})\right|}{\;\sqrt{\sum_{1}^{N}{\left|X(i,t)\right|}^{2}\cdot \sum_{1}^{N}{\left|Y(j,t)\right|}^{2}}}$	Nolte et al., 2004; **Dikker et al.**, **2021**
Envelope correlation	$r_{i,j}=\frac{\overset{N}{\underset{1}{\sum }}\left(nv_{x}-\bar{env_{x}}\right)\cdot \left(env_{y}-\bar{env_{y}}\right)}{\sqrt{\overset{N}{\underset{1}{\sum }}{\left(env_{x}-\bar{env_{x}}\right)}^{2}}\sqrt{\overset{N}{\underset{1}{\sum }}{\left(env_{y}-\bar{env_{y}}\right)}^{2}}}$	Mehrkanoon et al., 2014; Clerico et al., 2015; **Zamm et al.**, **2018a**
	*where env*_*x*_ = |*X*(*i, t*)|, *env*_*y*_ = |*Y*(*j, t*)|	
Power correlation	$r_{i,j}=\frac{\overset{N}{\underset{1}{\sum }}\left(amp_{x}-\bar{amp_{x}}\right)\cdot \left(amp_{y}-\bar{amp_{y}}\right)}{\sqrt{\overset{N}{\underset{1}{\sum }}{\left(amp_{x}-\bar{amp_{x}}\right)}^{2}}\sqrt{\overset{N}{\underset{1}{\sum }}{\left(amp_{y}-\bar{amp_{y}}\right)}^{2}}}\;$	Shaw, 1984; **Guevara and Corsi-Cabrera**, **1996**
	$where\;amp_{x}=|X\left(i,t\right){|}^{2},\;{\;amp}_{y}=|Y\left(j,t\right){|}^{2}\;$	
PLV	$r_{i,j}=\frac{1}{N}|\overset{N}{\underset{1}{\sum }}\varphi_{x}\cdot {\varphi_{y}}^{*}|$	Lachaux et al., 1999; **Dumas et al.**, **2010**
	$where\;\varphi_{x}=\frac{X\left(i,t\right)}{\left|X\left(i,t\right)\right|},\;{\;\varphi }_{y}=\frac{Y\left(j,t\right)}{\left|Y\left(j,t\right)\right|}$	
CCorr	$r_{i,j}=\frac{\overset{N}{\underset{1}{\sum }}sin\left(\theta_{x}-\bar{\theta_{x}}\right)\cdot sin\left(\theta_{y}-\bar{\theta_{y}}\right)}{\sqrt{\overset{N}{\underset{1}{\sum }}{sin\left(\theta_{x}-\bar{\theta_{x}}\right)}^{2}}\sqrt{\overset{N}{\underset{1}{\sum }}{sin\left(\theta_{y}-\bar{\theta_{y}}\right)}^{2}}}\;$	**Burgess**, **2013****; Goldstein et al.**, **2018**
	*where θ*_*x*_ = *angle*(*X*(*i, t*)), θ_*y*_ = *angle*(*Y*(*j, t*))	

*The table shows a list of connectivity measures/synchrony metrics implemented in the software. The references column lists relevant papers, with hyperscanning studies adopting the metric marked in bold. The equations are adapted from the first paper in the reference list. The result of the equation is one connectivity value r_(i, j)_ for electrode pair (i, j) in the N time-point window, computed from analytic signals X(i, t) and Y(j, t), as explained in section Connectivity Analysis*.

The metrics, which, as mentioned above, are a subset of those implemented in the hyperscanning analysis pipeline HyPyP (Ayrolles et al., 2021), include two variations of correlation (envelope and power correlation) and two of coherence (coherence and imaginary coherence) measures, which are traditional linear methods to estimate brain connectivity, as well as two measures of phase synchrony (PLV and CCorr). While correlation methods are predominantly employed in hyperscanning fMRI studies to characterize joint action and shared attention (Koike et al., 2016), as shown in the case study below, we have validated power correlation as a neurofeedback synchrony signal. Coherence is more commonly used in fNIRS and EEG hyperscanning studies (Liu et al., 2016; Dikker et al., 2017; Miller et al., 2019). Imaginary coherence, i.e., the imaginary part of the coherence, was developed in response to coherence's susceptibility to zero-lagged spurious synchrony, and has been found to reflect personality traits and social closeness in hyperscanning studies (Nolte et al., 2004; Dikker et al., 2021). For phase synchrony, we included PLV, which has been widely used in hyperscanning studies to capture joint action (Dumas et al., 2010), verbal interaction (Perez Repetto et al., 2017), decision-making (Tang et al., 2016) and other tasks. CCorr measures the covariance of phase variance between two data streams and is more robust to coincidental synchrony (Burgess, 2013) compared to PLV, and has been used to investigate touch (Goldstein et al., 2018), learning (Bevilacqua et al., 2019) and language (Perez Repetto et al., 2017) in hyperscanning studies.

In addition to the connectivity metric, users are also able to choose “connectivity type” (Figure 3), which determines the electrode pair combination for connectivity. For each frequency band, the computation is carried out for every possible electrode pair between the participants, and then averaged based on the “connectivity type” parameter. If “connectivity type” is “one-to-one,” only electrode pairs in the matching position are considered (e.g., Fp1 channel of participant A is only paired with Fp1 of participant B and C, etc.); alternatively, if it is set to “all-to-all,” all electrode pairs are considered in v the averaging.

Connectivity Analysis outputs data chunks to LSL as a “Marker” stream under the name “Rvalues.” The size of this data chunk depends on the number of subjects and the number of frequency bands chosen for analysis. For example, if there are 4 subjects and 4 frequency bands, the data chunk will be a vector of length 24 (6 combinations of pairs times 4 frequency bands). Additionally, if the checkbox “sending through OSC” is selected, the same data chunks will simultaneously be transmitted through the OSC protocol with parameters in “OSC IP address” and “OSC port.”

### Normalization

As part of Connectivity Analysis, normalization of connectivity values is implemented with two options: manual normalization (labeled as “Manual” in Figure 3) and baselining with a pre-recorded file (labeled as “from file” in Figure 3). With a Min-Max normalization method, the user can use either of the options, or a mixture of both with a weighting factor adjusted by the slider “Weight.” The minimum and maximum limits are then weighted between the “Manual” and “from file” options.

### Visualization and Sonification

#### Visualization

**Figure 6:**
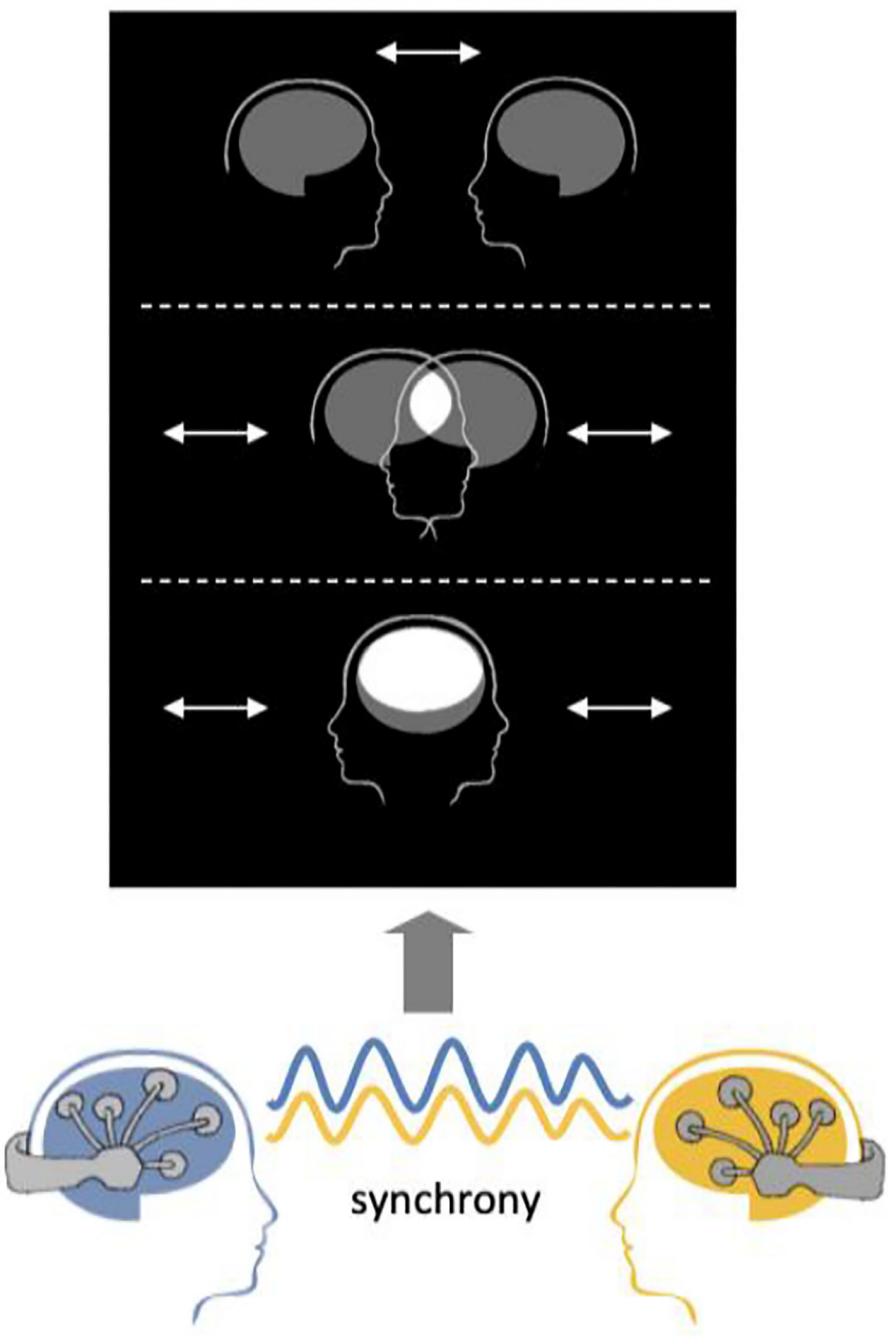
Visualization schematic. Connectivity values are visualized as the distance between the two merging heads using the visualization module.

The example visualization protocol provided with the software is based on *Mutual Brainwaves Lab* (Figure 6), described in Supplementary Material, Mutual Brainwaves Lab. The visualization app was originally built in C++ using the OpenFramework toolkit (https://openframeworks.cc/), a general-purpose framework that wraps together several libraries to assist the creative process. An updated Python3 version makes it easier to deploy the application on different OS (Windows, MacOS, Linux) and more maintainable and extendable for developers, thanks to Python's more accessible syntax and a wide choice of libraries. The application relies on an OSC plugin that listens in real-time for an OSC sender over the network. The GUI of the application is built using OpenFramework for the C++ version and PyQt5 (https://pypi.org/project/PyQt5/) for the Python version. When launched, it presents two avatars representing human heads with a brain icon, and a menu with various options to parameterize the interface. As soon as the OSC receiver starts receiving a stream of data from the Hybrid Harmony running on the same network, the application translates the inter-brain synchrony as the distance between the avatar heads. The user can also set up sessions where participants are encouraged to “score” higher synchrony in a limited timeframe.

#### Sonification

Sonification of EEG data has been explored in projects such as EEGsynth (https://github.com/eegsynth/eegsynth), which interfaces electrophysiological recordings (e.g., EEG, EMG and ECG) with analog synthesizers and digital devices. Here, we demonstrate that Hybrid Harmony can be easily interfaced with a digital audio workstation (DAW) in real-time through an OSC (Supplementary Material, The Data Transfer Protocol: Open Sound Control) plugin, allowing for the control of audio parameters based on the connectivity values sent through OSC. We describe the protocol for the control of Ableton Live via LiveGrabber (https://www.showsync.com/tools#livegrabber), a set of free Ableton plugins. LiveGrabber receives messages from any OSC sender on the network and uses OSC messages to control track parameters in Ableton.

After specifying the OSC IP address and port in the Hybrid Harmony GUI, the output can be received by the GrabberReceiver plugin in Ableton (part of the LiveGrabber package). The TrackGrabber plugin allows for the control of track parameters (such as volume, reverb, panning, etc.) using the output from Hybrid Harmony in real time. To illustrate a simple sonification example, we have created a soundscape in which the volume of certain musical pitches can be modulated to create alternating moments of *dissonance* (harmonic tension or unpleasant sounding chords) and *consonance* (harmonic resolution or pleasant, stable chords). The volume of each pitch is directly controlled by connectivity parameters output through OSC, such that greater connectivity values (moments of increased interpersonal synchrony) correspond to more pleasant, stable sounding chords.

## Validation

**Figure 7:**
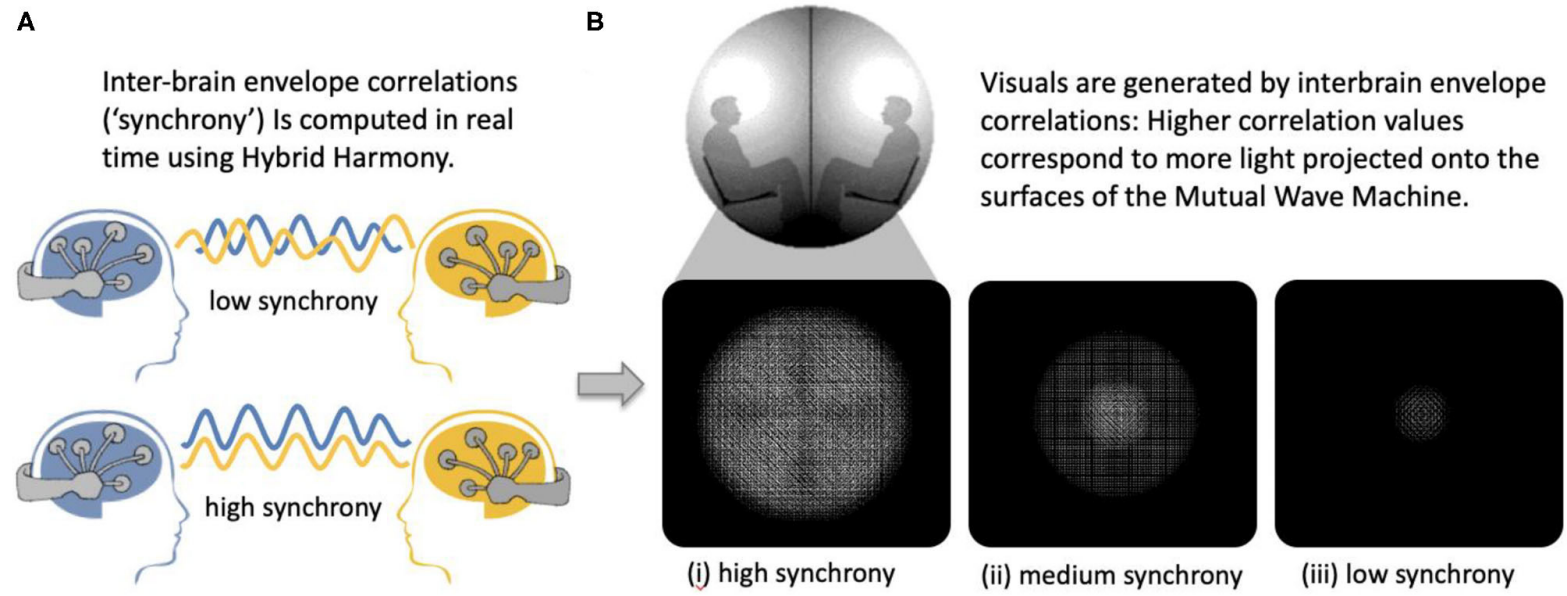
The Mutual Wave Machine using inter-brain envelope correlations with Hybrid Harmony. **(A)** Inter-brain correlations between two participants wearing wireless EEG headsets were computed in real time. **(B)** Higher inter-brain correlation values correspond to more light projected on each of the surfaces, with the focus point behind each participant's head.

To validate that Hybrid Harmony can capture socially relevant self-report measures, we used a dataset of 243 dyads participating in the Mutual Wave Machine (Figure 7; see Supplementary Material, Case study: The Mutual Wave Machine for details), during which real-time envelope correlations were recorded and translated into light patterns projected onto the surface of two spheres (Hybrid Harmony parameters: “Frequency bands for analysis”: delta 1–4 Hz, theta 4–8 Hz, alpha 8–12 Hz, beta 12–20 Hz; “Connectivity type”: one-to-one; “Connectivity metric”: envelope correlation; “Window size”: 3 s).

We first asked whether the average envelope correlation reflected dyads' personal distress (Davis, 1980), building on past research where we consistently find a negative relationship between personal distress and inter-brain synchrony (Dikker et al., 2017, 2021; Chen et al., 2021; Reinero et al., 2021). Indeed, we find that pairs' average personal distress was negatively correlated with their neurofeedback synchrony in theta [*r*_(236)_ = −0.182, pFDR = 0.010], alpha [*r*_(236)_ = −0.204, pFDR = 0.004] and beta [*r*_(236)_ = −0.178, pFDR = 0.010].

We then asked whether social closeness (Aron et al., 1992) was positively related to pairs' envelope correlations. We reasoned that this is an interpersonal state measure that might be the target for a social neurofeedback intervention. Indeed, we find significant positive correlations between social closeness and pairs' neurofeedback synchrony in the alpha [*r*_(236)_ = 0.264, pFDR <0.001] and beta bands [*r*_(236)_ = 0.210, pFDR = 0.004].

While these results do not speak to the efficacy of Hybrid Harmony as a social neurofeedback tool, they confirm a very important first step, namely that the neurofeedback output is correlated with socially relevant features that might be the target for neurofeedback interventions.

## Discussion

We describe Hybrid Harmony, an open-source software that allows researchers to explore interpersonal synchrony in a plug-and-play setup. The project builds on previous work from our group suggesting that incorporating synchrony neurofeedback in naturalistic social interactions may help increase synchrony and interpersonal connectedness, as such raising the possibility that biofeedback may constitute a useful tool to explore meaningful features of social interaction (Dikker et al., 2019, 2021).

As discussed in the Introduction, inter-brain synchrony has been shown to correlate with a range of personal and social characteristics and behaviors, underscoring its relevance in understanding naturalistic social interactions. Interpersonal biofeedback approaches may make it possible to more precisely map such social and psychological factors onto specific neurophysiological processes. For example, testing different types of synchrony in a neurofeedback environment might help inform the field about which metric is the most indicative of social behavior and outcomes in which social contexts. Dyads may be more responsive to multi-brain neurofeedback based on, say, coherence during collaborative tasks, but more responsive to, say, envelope correlations during social sharing. Future findings of such a nature will enrich our knowledge about the social relevance of these metrics and constitute a non-invasive way to probe possible causal links between inter-brain synchrony and social behavior (Moreau and Dumas, 2021; Novembre and Iannetti, 2021). Here we show in a dataset of 243 dyads that social closeness and affective traits can, in fact, be reflected in online synchrony neurofeedback measures, which is an important firspage t step in this direction.

### Future Directions and Challenges

Beyond inter-brain coupling, interpersonal synchrony has been examined in more depth in other aspects of behavior, including movement (Oullier et al., 2008; van Ulzen et al., 2008; Varlet et al., 2011), language (Pickering and Garrod, 2004) and physiological rhythms such as heart rate and respiration (Müller and Lindenberger, 2011; Noy et al., 2015). As described above, movement and physiological synchrony may be both cause and effect of inter-brain coupling. In future iterations of Hybrid Harmony, we hope to extend the software to incorporate multiple data streams including physiological and movement data. This would allow users to compare the social relevance of various forms of synchrony, and possibly to tease apart interrelationships between (neuro)physiological and behavioral coupling (e.g., Dumas et al., 2010; Mayo and Gordon, 2020; Pan et al., 2020). Similarly, while we prioritized EEG research in Hybrid Harmony, given the increasingly rich fNIRS hyperscanning literature (Liu et al., 2016; Nozawa et al., 2016; Miller et al., 2019) and recent successful work in fNIRS neurofeedback (Gvirts and Perlmutter, 2020; Kohl et al., 2020) we believe an extension of Hybrid Harmony to include metrics suitable for fNIRS data would be a very welcome and fruitful future direction for the software.

EEG systems' susceptibility to movements and environmental noises can greatly compromise data quality and introduce spurious synchrony in our measure. In controlled lab studies, motion artifacts are often carefully removed manually and through data decomposition (e.g., principal component analysis). In the neurofeedback setting, however, such procedures haven't been widely implemented. In our practice, we tried to address the issue empirically with several solutions. For example, we piloted a version of the Mutual Wave Machine where sudden motion-related fluctuations in the data were removed, but this dramatically influenced the experience: participants often react enthusiastically to a sudden increase in light, only to get “punished” for facial expression, which would discourage them from naturally engaging with each other. We considered patching the data with correlations from non-contaminated stretches of data, but this would lead to arbitrary choices. We therefore instead opted for an alternative solution where participants were told explicitly that because extensive head and facial movements can dramatically affect the EEG signal, what they were seeing could also be caused by synchronous noise or synchronous movement. While this option sufficed for the experiential side of the neurofeedback, it is suboptimal with regard to data fidelity. In future releases of the software, we will incorporate support for online data cleaning procedures such as toolboxes (Mullen et al., 2015) and EEG systems that provide built-in data cleaning options in their software, e.g., the SMARTING system by mBrainTrain (Lee et al., 2020).

In addition to challenges related to data cleaning, the real-time nature of the analysis procedure poses challenges in terms of its interpretation. For instance, data fidelity is much higher when applying filtering and correlation analysis on larger stretches of data, but in the type of analysis employed here, this would compromise the immediacy of the neurofeedback. Therefore, it is important to note that our real-time approach might not be able to characterize those types of synchrony that may not be temporally aligned over short intervals. For example, fMRI studies have suggested there may be delays of up to 8 seconds in inter-brain synchrony between speakers and listeners (Stephens et al., 2010a; Dikker et al., 2014; Misaki et al., 2021). Indeed, while successful joint action is typically associated with the coupling of motor movements (Dumas et al., 2010), being “in sync” or “on the same wavelength” is often taken to imply interactive alignment at the level of mental representations (Garrod and Pickering, 2009; Pickering and Garrod, 2013), usually involving more “abstract” constructs such as sharing viewpoints (Van Berkum et al., 2009). These mental representations may or may not be linked to convergence at the temporal level. In line with this dissociation, in two instances of the Mutual Wave Machine we asked participants to reflect on their “connection strategies.” Pairs who used either eye contact or joint action as a connection strategy (mimicry, laughter, motion coordination) exhibited an increase in inter-brain synchrony over time as measured by Imaginary Coherence and Projected Power Correlation (Dikker et al., 2021). Such an increase in synchrony was not observed for pairs who tried “thinking about the same thing.” While these results validated our approach in capturing synchrony in joint action, they do not exclude the possibility of synchrony on the abstract mental representations, given that such synchrony may entail more complex temporal dynamics.

In future iterations, we hope to incorporate alternatives to the time-aligned approach, such as introducing a temporal delay between the data streams and real-time adaptive normalization. These additions will hopefully increase sensitivity to endogenous synchronizers and facilitate more controlled experimental designs (Stephens et al., 2010b).

Finally, it is worth reiterating that very little is known about the correspondence between different synchrony analysis metrics and socio-psychologically relevant factors. Although some metrics are better than others in theory (e.g., CCorr is more robust to spurious synchrony than PLV), and some are more common in hyperscanning studies than single brain studies (e.g., phase synchrony is more common than coherence/correlation), the exact pros and cons of each metric require further investigation. As more becomes known about the mapping between psychological processes as inter-brain synchrony metrics, we will add guidance for users with respect to the choice of metrics in different situations.

## Conclusion

In this study, we describe the background, functionality, and validation of Hybrid Harmony, a multi-person neurofeedback application for interpersonal synchrony. With its user-friendly interface and flexible design, Hybrid Harmony enables researchers to explore the interplay between synchrony as a computational method and the various psychological, cognitive, and social functions potentially associated with it.

## Data Availability Statement

The datasets presented in this study can be found in online repositories. The names of the repository/repositories and accession number(s) can be found at: https://github.com/RhythmsOfRelating.

## Ethics Statement

Ethical review and approval was not required for the study on human participants in accordance with the local legislation and institutional requirements. Written informed consent to participate in this study was provided by the participants' legal guardian/next of kin. Written informed consent was obtained from the individual(s), and minor(s)' legal guardian/next of kin, for the publication of any potentially identifiable images or data included in this article.

## Author Contributions

SD, MO, and PC contributed to conception and design of the study. MO, DM, SH, MR, GD, and PC developed the software. PC performed the statistical analysis. PC, SD, SH, MR, KS DM, and GD wrote sections of the manuscript. All authors contributed to manuscript revision, read, and approved the submitted version.

## Conflict of Interest

DM is the co-director of Diademics Pty Ltd company. The handling Editor declared a shared affiliation, though no other collaboration, with one of the authors MR. The remaining authors declare that the research was conducted in the absence of any commercial or financial relationships that could be construed as a potential conflict of interest.

## Publisher's Note

All claims expressed in this article are solely those of the authors and do not necessarily represent those of their affiliated organizations, or those of the publisher, the editors and the reviewers. Any product that may be evaluated in this article, or claim that may be made by its manufacturer, is not guaranteed or endorsed by the publisher.
