# Supplementary material for: Hybrid Harmony: A Multi-Person Neurofeedback Application for Interpersonal Synchrony
Source: Front Neuroergon. 2021 Aug 12;2:687108. doi: 10.3389/fnrgo.2021.687108 (PMC10790844; doi:10.3389/fnrgo.2021.687108)
Supplement: Supplementary file 1 [file Data_Sheet_1.pdf]

Supplementary Material

S1 Mutual Brainwaves Lab

Mutual Brainwaves Lab is an interactive neurofeedback game that tracks and visualizes inter-brain synchrony as two heads merging in and out of each other. Whenever "synchrony" meets a certain threshold, the heads perfectly overlap. Mutual Brainwaves Lab has been implemented as a neuroscience educational and outreach tool. In some versions, the interface also displays a countdown timer and a score that goes up every time the heads overlap perfectly. As such, participants are challenged to get the "highest" synchrony score during e.g., a 3-minute period (a clock counts down the seconds on the display). In ***NeuroTango*** (Chen, 2014), the Mutual Brainwaves Lab interface was used during an evening event discussing social connections in tango dance. Two pairs of tango dancers competed against each other for the highest synchrony score during different "conditions": dancing with familiar partners, switching partners, dancing to music, dancing without music, listening to music without dance, and perfect silence. While the data was not used for scientific purposes, the neurofeedback environment served as a tangible illustration and conversation-starter for a discussion surrounding the neuroscientific constructs: synchrony during joint action, entrainment, and emotional effects of connectedness during dance (see also Basso et al., 2021; Dikker et al., 2019; Goede et al., 2022). Additional explorations into synchrony in the arts include ***Ondas***, where musician Residente (René Pérez Joglar) and Bad Bunny (Benito Antonio Martínez Ocasio) used the Mutual Brainwaves Lab to seek connectedness via shared interests and shared musical memories (www.youtube.com/watch?v=kJBlSIZmGNA&t=50s; Associated Press, 2019).

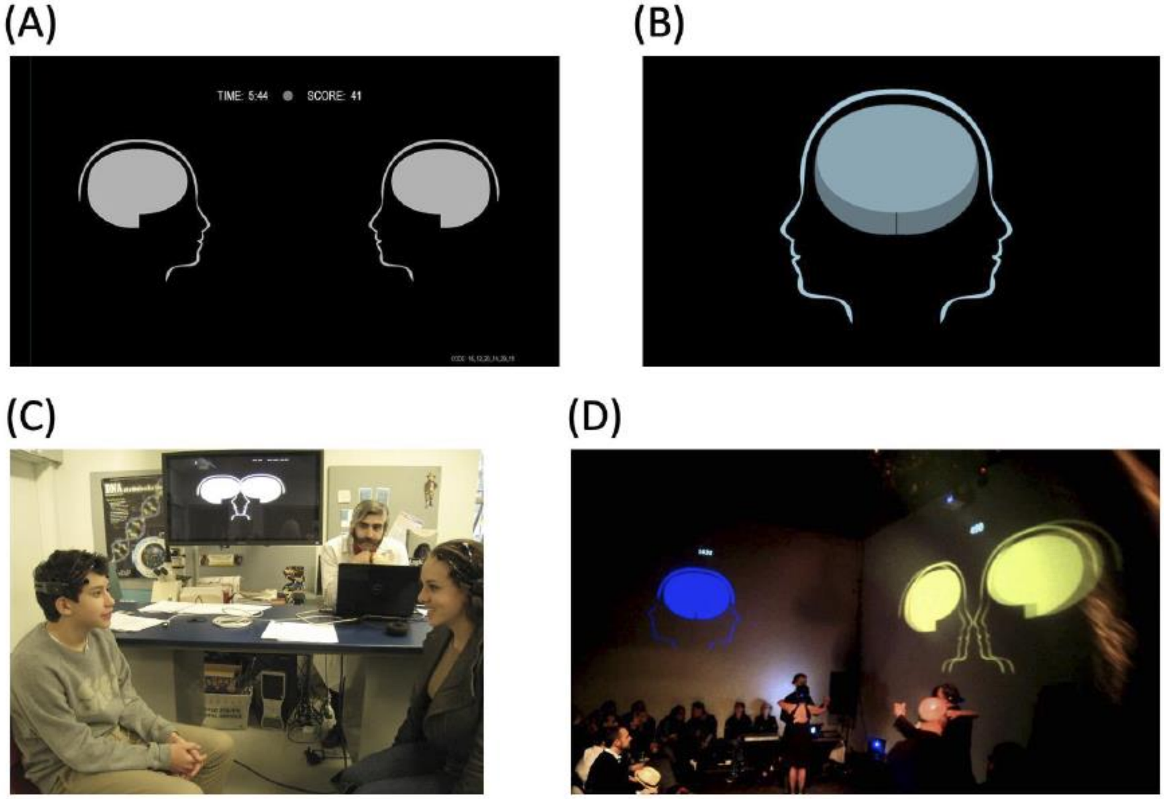

Supplementary Figure S1. Mutual Brainwaves Lab

Venues include the American Museum of Natural History (New York City, 2013), World Science Festival (New York City, 2013, 2014); Basilica Hudson (Hudson, 2014), Pioneer Works (Brooklyn, 2016), among others). (**A/B**) View of Mutual Brainwaves Lab display with no synchrony (A) and "full" synchrony (B). See www.youtube.com/watch?v=d64SeneJpgY for rendering. **(C)** Impression from the American Museum of Natural History (2013). Photograph courtesy Ellen Pearlman. **(D)** Still from NeuroTango performed at La Sala Brooklyn (2014).

S2 Case study: The Mutual Wave Machine

We tested the efficacy of the neurofeedback component by correlating the synchrony values that were fed to the Mutual Wave Machine visualization directly with self-report measures. Specifically, following up on our previous findings (Dikker et al., 2021), we analyzed a new Mutual Wave Machine dataset and asked whether the online synchrony values using the Hybrid Harmony protocol were correlated with pairs' Personal Distress and Social Closeness. Offline analyses on this same dataset are reported in Chen, Kirk, and Dikker (2021).

In the **Mutual Wave Machine**, two visitors are enclosed by an intimate capsule and immersed in an audiovisual environment that responds to and reflects their shared brain activity. During the ~10-minute experience, dyads are invited to engage in face-to-face social interaction and explore their efforts to approach or distance themselves from each other.

Over 4,000 people have participated in the Mutual Wave Machine to date across more than a dozen sites (see wp.nyu.edu/mutualwavemachine for details). Results from ~ 1,500 participants (Dikker et al., 2021) suggest that empathy, mood and social connectedness are strong predictors of inter-brain synchrony (computed offline and quantified using imaginary coherence, Nolte et al., 2004; and projected power correlations, Hipp et al., 2012). For example, replicating findings from Dikker et al. (2017), pairs of participants with high Personal Distress (a tendency to experience discomfort in response to others' distress) exhibited less synchrony than those with low Personal Distress personalities (see also Goldstein et al., 2018 for results showing that empathy affects brain-to-brain synchrony). Similar effects were observed for social connectedness using the Inclusion of the Other in the Self Scale (Aron et al., 1992). More recently, Chen, Kirk and Dikker (2021) also reported significant correlations between mindfulness measured by Mindful Attention Awareness Scale (MAAS; Kotzé & Nel, 2016a, 2016b) and inter-brain synchrony computed offline using Circular Correlation Coefficient (CCorr; Lachaux et al., 1999; Pérez et al., 2019).

To date, the Mutual Wave Machine (DIKKER+OOSTRIK, 2013-ongoing) has been exhibited at *TodaysArt Festival* (The Hague, 2013), *Eye Film Institute* (Amsterdam, 2013), *Lexus Hybrid Art* (Moscow, 2014), *Nemo Science Center* (Amsterdam, 2014), *Lowlands Festival* (The Netherlands, 2015), *Benaki Museum* (Athens Greece, 2016), *3LD Art & Technology Center* (New York City, 2016), *FORMS Festival* (Toronto, 2016), *Pioneer Works* (Brooklyn, 2017), *OPUS 1*, *Merriweather* (Maryland, 2017), *Espacio Fundación Telefónica* (Madrid, 2019), *Museu Nacional de Arte Contemporânea* (Lisbon, 2021), among others. EEG was recorded using the EMOTIV EPOC (reported in Dikker et al., 2021), or the MUSE headband (Madrid, reported here and in Chen et al., 2021). A video documentation can be viewed here: https://vimeo.com/96287858.

S2.1 Participants

676 individuals (338 pairs) participated in the study (356 females, 318 males; 2 participants identified as "other"). Participants' age ranged from 15 to 88 years old (mean age: 35.4) Participants completed the questionnaires and consent forms in Spanish. Individual written informed consent was obtained before the experimental session. There was no monetary compensation for participation.

S2.2 Experimental Setup

In this study, we collected data at the participatory art installation Mutual Wave Machine, from people who participated at Espacio Telefónica in Madrid, Spain (2019) from October 31st, 2018 to January 9th, 2019. Among iterations of this experiment, we use data from this site because other iterations did not use the current version of the introduced tool Hybrid Harmony. Pairs of museum visitors were invited to engage in face-to-face interaction inside the Mutual Wave Machine for 10 minutes while wearing the MUSE headset, a four-electrode wireless EEG system (Krigolson et al., 2017). Real-time interbrain power correlation (see table 2) was calculated and used to generate visual neurofeedback projected onto the surface of the Mutual Wave Machine in the form of growing and shrinking moiré patterns. This resulted in a visual mapping where more synchrony was reflected in more light.

Participants were asked to complete a short questionnaire both before and after the session, addressing their relationship to each other and affective personality traits, including questions about relationship duration and social closeness. Social closeness was measured using the Inclusion of the Other in the Self Scale (Aron et al., 1992) and affective personality traits were assessed using the Interpersonal Reactivity Index (Davis & Others, 1980; e.g., "When I see someone who badly needs help in an emergency, I go to pieces"). In the current case study, we only examined Social Closeness and Personal Distress because here we only aim to demonstrate neurofeedback signals' validity in reflecting the social experience. We thus chose the two traits that have shown a robust trend in the offline analyses, but this choice does not constrain the possibility of further analyses.

S2.3 Materials

Both before and after the session, pairs of participants completed a questionnaire regarding their relationship with each other and their affective personality traits. Pairs' social closeness was assessed using the Inclusion of Other in the Self Scale (Aron, Aron, and Smollan 1992). Affective personality traits, including Personal Distress (e.g., "When I see someone who badly needs help in an emergency, I go to pieces") and Empathic Concern (e.g., "I often have tender, concerned feelings for people less fortunate than me"), was assessed with a revised 14-item version of the Interpersonal Reactivity Index (Davis & Others, 1980) on a five-point Likert scale ranging from "Does not describe me well" to "Describes me very well". After the session, as a mood measure, we also asked participants to complete a shortened version of the Positive and Negative Affect Schedule (PANAS-X; Watson & Clark, 1994).

S2.4 Data Analysis

To investigate the relationship between the online neurofeedback signal and pairs' Personal Distress and Social Closeness, we first processed the neurofeedback recordings and questionnaire data and removed participants with incomplete data, resulting in 243 pairs from the initial 338 pairs.

The neurofeedback recordings contained synchrony values for timepoints of the entire recording, divided into 1 Hz frequency bins ranging from 1 to 16 Hz. We averaged the

correlation values across all timepoints and within each frequency band (delta: 1-4 Hz, theta: 4-8 Hz, alpha: 8-12 Hz, beta: 12-16 Hz). We then removed data points that deviated from the mean more than 3 standard deviations, leaving 236 pairs for the next step.

We performed Pearson correlations between the neurofeedback values and Personal Distress and Social Closeness, corrected for multiple comparisons using False Discovery Rate (FDR; Benjamini & Hochberg, 1995). We averaged Personal Distress and Social Closeness scores for each pair, measured before the experience for each pair. Although both traits were also measured after the experience, we only chose the ones before the session in order to match the approach in offline analysis of similar datasets (Dikker et al. 2021).

S2.5 Results

We found significant correlations between social closeness and pairs' neurofeedback synchrony in the alpha ($r(236) = 0.264$, $pFDR < 0.001$) and beta bands ($r(236) = 0.210$, $pFDR = 0.004$). Pairs' average Personal Distress was negatively correlated with their neurofeedback synchrony in theta ($r(236) = -0.182$, $pFDR = 0.010$), alpha ($r(236) = -0.204$, $pFDR = 0.004$) and beta ($r(236) = -0.178$, $pFDR = 0.010$) bands.

Traits	Freq. bands	Corr. Coef.	p	pFDR
Personal Distress	delta	-0.006	0.929	0.929
	theta	-0.182	0.005	**0.010**
	alpha	-0.204	0.002	**0.004**
	beta	-0.178	0.006	**0.010**
Social Closeness	delta	-0.107	0.101	0.115
	theta	0.124	0.057	0.076
	alpha	0.264	0.000	**<0.001**
	beta	0.210	0.001	**0.004**

Supplementary Table S1. Pearson Correlation between affective traits and synchrony
The first two columns are variables for the Pearson Correlation. The numerical columns represent correlation coefficients, p values without correction for multiple comparisons, and p values after False Discovery Rate (FDR) correction. Significant results are marked as bold in the pFDR column.

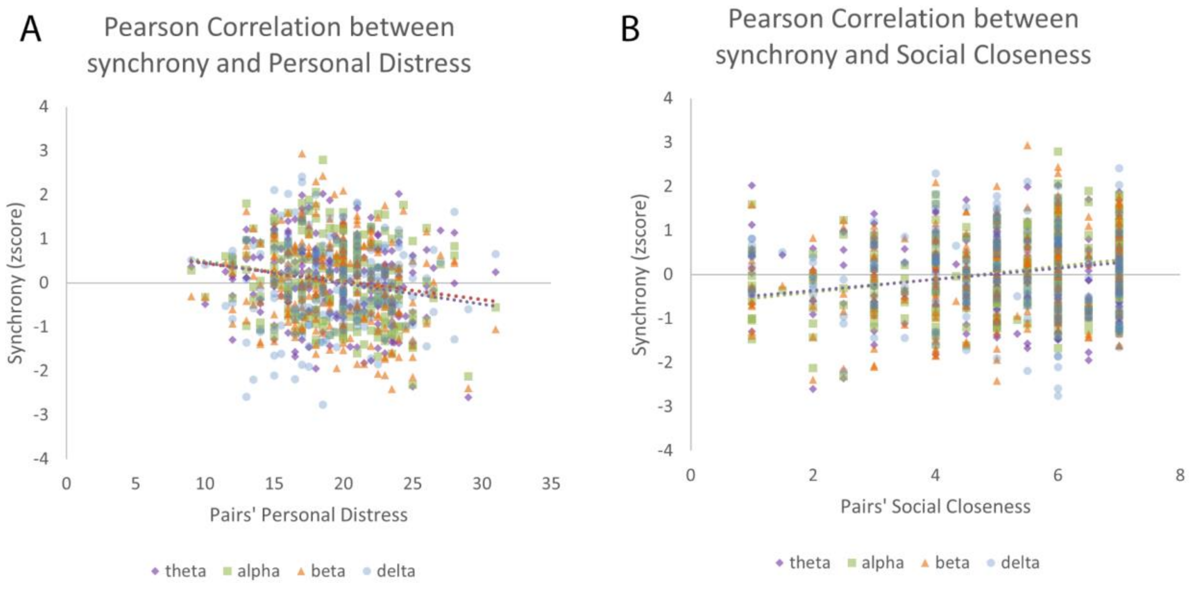

Supplementary Figure S2. Regression plots between affective traits and synchrony

(A) Pairs' average Personal Distress negatively predicts synchrony at theta ($r(236) = -0.182$, pFDR = 0.010), alpha ($r(236) = -0.204$, pFDR = 0.004) and beta ($r(236) = -0.178$, pFDR = 0.010) bands. **(B)** Pairs' average Social Closeness positively predicts synchrony at alpha ($r(236) = 0.264$, pFDR < 0.001) and beta bands ($r(236) = 0.210$, pFDR = 0.004).

S3 Data Transfer Protocol

We used the following third-party data transfer protocols for Hybrid Harmony, LabStreamingLayer and Open Sound Control, corresponding to 'LSL' and 'OSC' in figure 3 in the main text.

S3.1 The Data Transfer Protocol: LabStreamingLayer

To stream data from hardware devices to Hybrid Harmony, and to stream output results from Hybrid Harmony to any visualization or sonification software, we use the LabStreamingLayer (LSL; https://github.com/sccn/labstreaminglayer). LSL is a general purpose platform for online and offline data streaming and synchronization designed for mobile brain/body imaging experiments (Makeig et al., 2009) in particular, but for many more general

purpose applications as well. It was developed at the Swartz Center for Computational Neuroscience at University of California San Diego for use in multi-modal neurophysiological and behavioral experiments and is now a community supported open source software project with contributors world-wide. LSL is a widely used platform for data streaming and synchronization of millisecond per sample digitized biosignals.

LSL is comprised of a data streaming protocol, an underlying C/C++ based library that implements that protocol, wrapper libraries for many popular programming languages (including Python, C#, Java, and Rust), an ecosystem of opensource apps for connecting with various hardware devices, and a cross-platform recording app called LabRecorder that uses the XDF data format (https://github.com/sccn/xdf) to record data and timestamp information.

In addition to the opensource apps that connect hardware to LSL streams, many hardware vendors choose to native support LSL (which is MIT licensed) in their provided software packages. A current list of supported devices is available on the LSL readthedocs page: https://labstreaminglayer.readthedocs.io/info/supported_devices.html. In short, any EEG device that is LSL enabled may be used as an input to Hybrid Harmony.

1. S3.1.1 Stream discovery through LSL

LSL sits on top of a TCP/IP stack and provides methods for automated stream discovery. If, for example, an EEG device is connected to an LSL "outlet" (LSL parlance for mini-data server that forwards hardware data via the LSL network protocol), an LSL client (such as Hybrid Harmony) can probe the local area network and automatically discover any LSL outlet with the label "type EEG" ("type" and "EEG" are standard tokens for stream discovery in LSL—there are many others: https://labstreaminglayer.readthedocs.io/info/user_guide.html), and this is precisely what Hybrid Harmony does. The GUI displays available EEG streams and the user may choose which ones with which to connect for synchrony analysis.

2. S3.1.2 Using LSL for output

Hybrid Harmony may also use LSL to broadcast the output of its analysis. Values corresponding to the real-time output of synchronization metrics may be output in their own LSL streams that emanate from inside Hybrid Harmony. These values can be used in real-time to control visualization or sonification software provided they also utilize LSL.

3. S3.1.3 Saving data through LSL

LSL has an accompanying recording program LabRecorder (https://github.com/labstreaminglayer/App-LabRecorder), which allows for recording all available LSL streams on the same local network with synchronized timing. Thus, if desired, EEG input data as well as analysis outputs from Hybrid Harmony may be recorded in the XDF file format for offline analysis or other purposes. This makes Hybrid Harmony useful for researchers who wish to validate and/or run further analysis on the harvested EEG data and synchrony metric outputs.

S3.2 The Data Transfer Protocol: Open Sound Control

In addition to LSL, Open Sound Control (http://opensoundcontrol.org/; Freed & Schmeder, 2009) may be used as an online messaging system for broadcasting the output of Hybrid Harmony. OSC is a convenient option as it has wide-scale adoption in many platforms for computer sound and video synthesis including digital audio/visual workstations such as Ableton Live and Resolume, as well as audio/visual programming environments and frameworks such as Pure Data/GEM, Max/MSP/Jitter, OpenFrameworks, and many others. OSC was used in the visualization and sonification modules of Hybrid Harmony.
